# Clustering of clinical symptoms using large language models reveals low diagnostic specificity of proposed alternatives to consensus mast cell activation syndrome criteria

**DOI:** 10.1016/j.jaci.2024.09.006

**Published:** 2024-09-13

**Authors:** Benjamin D. Solomon, Purvesh Khatri

**Affiliations:** 1 Division of Allergy and Immunology, Department of Pediatrics, Stanford University, Palo Alto, CA, USA; 2 Institute for Immunity, Transplantation and Infection, School of Medicine, Stanford University, CA 94305; 3 Center for Biomedical Informatics Research, Department of Medicine, School of Medicine, Stanford University, CA 94305

**Keywords:** Mast cell, Mast cell activation syndrome, Mastocytosis, Anaphylaxis, Artificial intelligence, Large language model, Natural language processing, Generative pretrained transformer

## Abstract

**Background:**

The rate of diagnosis of mast cell activation syndrome (MCAS) has increased since the disorder’s original description as a mastocytosis-like phenotype. While a set of consortium MCAS criteria is well described and widely accepted, this increase occurs in the setting of a broader set of proposed alternative MCAS criteria.

**Objective:**

Effective diagnostic criteria must minimize the range of unrelated diagnoses that can be erroneously classified as the condition of interest. We sought to determine if the symptoms associated with alternative MCAS criteria result in less concise or consistent diagnostic alternatives, reducing diagnostic specificity.

**Methods:**

We used multiple large language models, including ChatGPT, Claude, and Gemini, to bootstrap the probabilities of diagnoses that are compatible with consortium or alternative MCAS criteria. We utilized diversity and network analysis to quantify diagnostic precision and specificity compared to control diagnostic criteria including systemic lupus erythematosus (SLE), Kawasaki disease, and migraines.

**Results:**

Compared to consortium MCAS criteria, alternative MCAS criteria are associated with more variable (Shannon diversity 5.8 vs. 4.6, respectively; p-value=0.004) and less precise (mean Bray-Curtis similarity 0.07 vs 0.19, respectively; p-value=0.004) diagnoses. The diagnosis networks derived from consortium and alternative MCAS criteria had lower between-network similarity compared to the similarity between diagnosis networks derived from two distinct SLE criteria (cosine similarity 0.55 vs. 0.86, respectively; p-value=0.0022).

**Conclusion:**

Alternative MCAS criteria are associated with a distinct set of diagnoses compared to consortium MCAS criteria and have lower diagnostic consistency. This lack of specificity is pronounced in relation to multiple control criteria, raising the concern that alternative criteria could disproportionately contribute to MCAS overdiagnosis, to the exclusion of more appropriate diagnoses.

## CAPSULE SUMMARY

Using a novel application of large language models, the authors demonstrate that proposed alternative definitions of MCAS are significantly less specific to mast cell activation than consortium MCAS criteria, potentially contributing to MCAS overdiagnosis.

## INTRODUCTION

Mast cell activation syndrome (MCAS) originated as a term to describe the phenotype of mast cell activation (MCA) in patients with features of systemic mastocytosis. Sonneck *et al*. used the term to unify patients with profound hypotension in response to hymenoptera envenomation who either fully or partially met systemic mastocytosis criteria^1^. Akin *et al.* used mast cell activation to describe patients with recurrent idiopathic anaphylaxis with or without features of monoclonal mast cell disease^2^. Throughout multiple iterations, MCAS consensus criteria have consistently required (i) the presence of severe, systemic, and recurrent symptoms associated with mast cell degranulation (e.g. anaphylaxis), in addition to (ii) evidence of acute mast cell mediator elevation above a patient-specific baseline and (iii) therapeutic response to mast cell-directed therapies^3–6^. The European Competence Network on Mastocytosis and the American Initiative in Mast Cell Diseases (ECNM-AIM) consortium criteria (hereafter designated “consortium MCAS criteria”) reaffirm this definition and further expand on the relation of MCAS to the broader MCA disorders (MCADs), including monoclonal (e.g. clonal mast cell disease with activating mutations in c-KIT), secondary (e.g. severe IgE-mediated hypersensitivities), and idiopathic MCAS ^7^.

However, alternatives definitions for MCAS have been proposed outside of expert organizations like ECNM and AIM and utilized as unvalidated clinical criteria (hereafter designated “alternative MCAS criteria”) ^8^. The symptoms accepted by these proposed alternative criteria are more numerous than those of the consortium MCAS criteria (153 vs. 18, respectively), invoke a broader range of clinical systems, appear less specific to the physiologic effects of mast cell mediators, and do not require the severity of consortium MCAS criteria. Laboratory criteria can be satisfied by isolated mast cell mediator elevation above a reference range, rather than acute elevation above a patient-specific baseline. However, baseline mast cell mediator levels exhibit significant inter- and intra-personal variation^9–11^, limiting laboratory specificity and increasing the relative influence of symptoms alone in satisfying alternative criteria compared to consortium criteria.

Patients referred to allergists for idiopathic MCAS often do not demonstrate abnormal mast cell activation. In a study of MCAS referrals, only 3% of patients demonstrated elevated tryptase levels during acute episodes and only 21% achieved symptom control with mast cell-directed therapies^12^, suggesting an alternative pathophysiology. Importantly, 56% of referrals were based on patient self-evaluation and internet research, which are unlikely to be accompanied by supportive laboratory data due to the specialized nature of mast cell mediator testing and interpretation.

Due to the disproportionate weight of symptomatology in alternative MCAS criteria and the factors that motivate referrals for evaluation of MCAS, we sought to address whether differences in symptom-based criteria between consortium and alternative MCAS criteria could contribute to overdiagnosis. We utilized multiple transformer-based large language models (LLMs), including ChatGPT, Claude, and Gemini, to evaluate criteria specificity based on symptom-diagnosis associations. These LLMs model complex semantic relationships to derive computational understanding of natural language, are capable of numerous natural language tasks within medicine^13^, and have been shown to encode clinical knowledge within their internalized embeddings^14^. Here, we utilized these LLMs to generate diagnosis probability distributions to evaluate MCAS criteria specificity.

## RESULTS AND DISCUSSION

**Figure 1:**
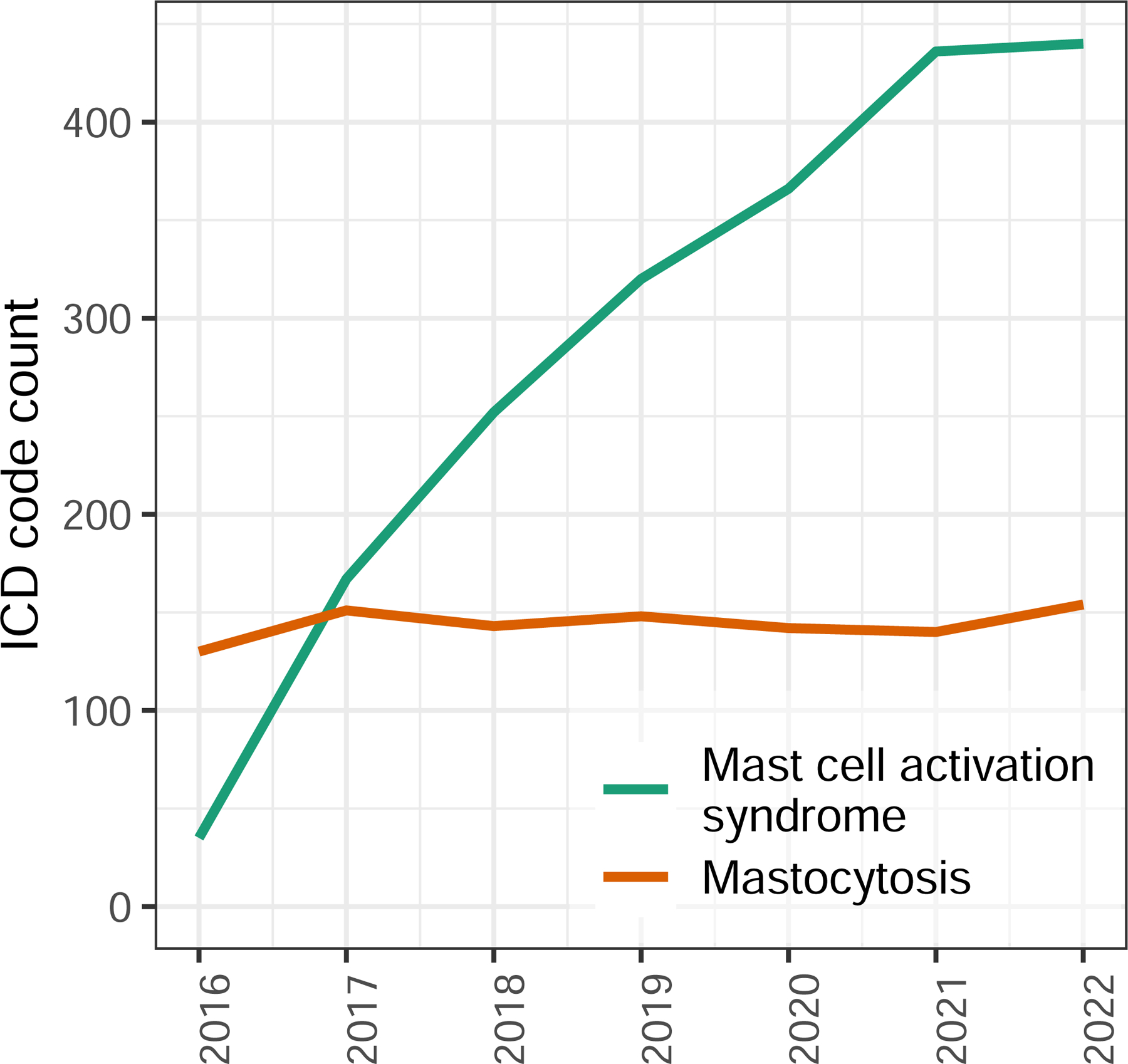
California ICD code use for inpatient encounters. Total count of indicated diagnosis codes per year.

Increasing referrals for idiopathic MCAS have been anecdotally reported. Using California ICD code data for inpatient admissions, we quantified the change in MCAS diagnosis rates over time. Since the creation of its ICD code in 2016, the rate of idiopathic MCAS increased 12.6-fold, while the rate of mastocytosis remained constant (Figure 1). While patients with systemic mastocytosis outnumbered those with idiopathic MCAS in the original case series, the annual number of admissions involving systemic mastocytosis was surpassed by idiopathic MCAS within 1 year of the latter’s inclusion in the ICD-10.

**Figure 2:**
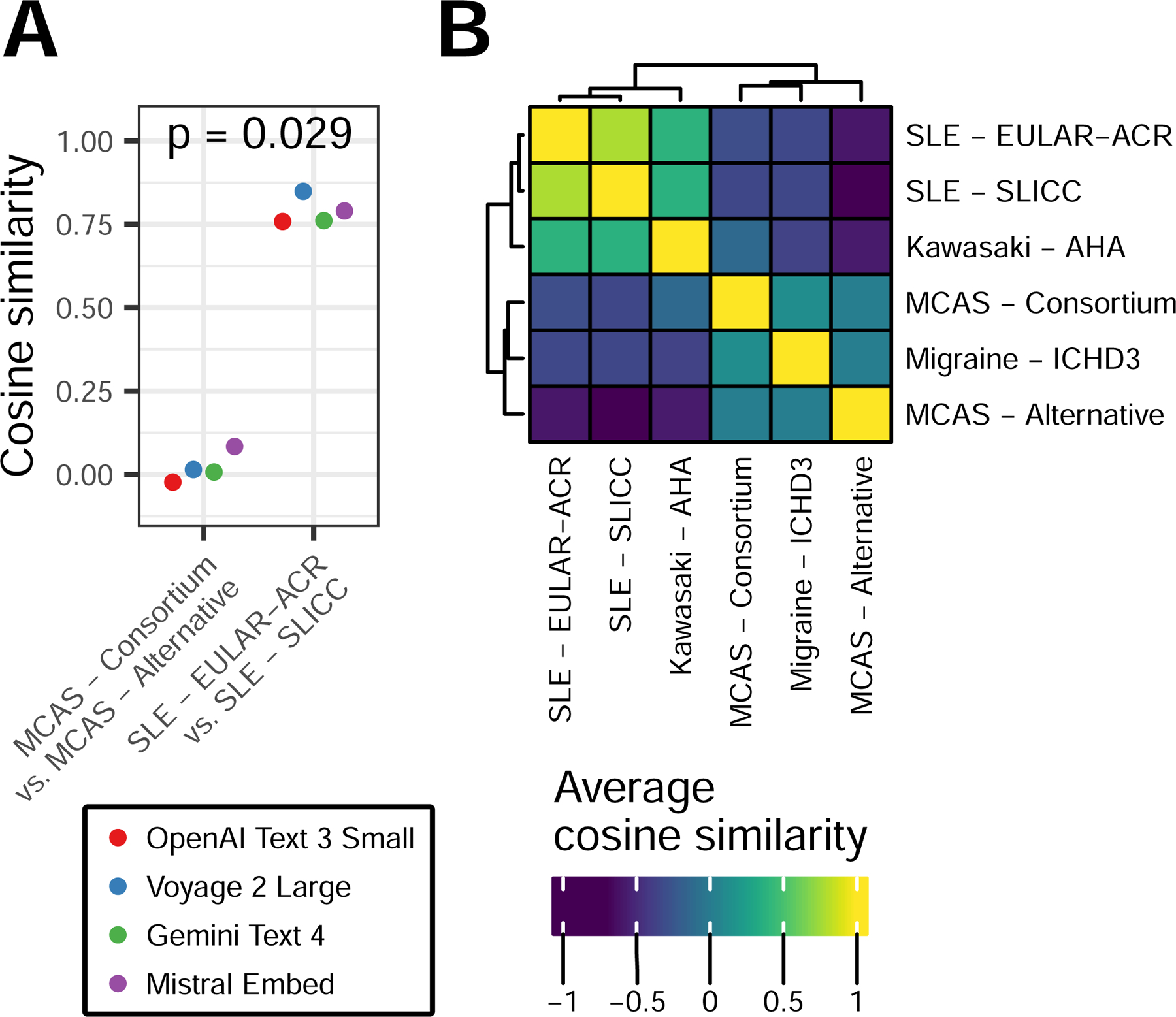
Similarity of diagnostic criteria based on symptom word embeddings. Multiple word embedding models were used to obtain embeddings for all symptoms within each set of criteria. Embeddings were then reduced by PCA. **A)** Cosine similarity between the PCA-embedding centroids of the indicated pairs of criteria. Colors represent individual embedding models, p-value by Wilcox rank sum test. **B)** As in **A** but averaged across all models to show similarity between all pairs of criteria.

For subsequent comparisons of consortium- and alternative-MCAS criteria, we identified several sets of control criteria that also emphasize patient symptoms, including AHA Kawasaki disease criteria^15^, EULAR-ACR^16^ and SLICC^17^ systemic lupus erythematosus (SLE) criteria, and ICHD-3 criteria for migraine with aura^18^. The latter was included to ensure that any differences observed in our results were not simply due to differences between inflammatory conditions. Across multiple word embedding models, which quantify semantic similarity of simple words or phrases, we observed that the semantic similarity between the two MCAS criteria was significantly lower than that between the two SLE criteria (mean cosine similarity 0.2 vs. 0.79, respectively; p-value=0.024; Figure 2A-B).

To maximize specificity and avoid false positives, the range of conditions that can satisfy a set of diagnostic criteria must be limited. We quantified the extent to which symptoms in consortium- and alternative-MCAS criteria converge on a set of possible diagnoses by simulating differential diagnoses using several LLMs, including ChatGPT-3.5, ChatGPT-4.0, Claude3-Haiku, Claude3-Opus, Gemini-1.0-Pro, and Gemini-1.5-Pro. Using a random subsample of symptoms from each set of criteria, we queried each LLM to build a ten-item differential diagnosis. We repeated this process 10,000 times to bootstrap a diagnosis probability distribution for each set of criteria. Compared to word embedding models, LLMs capture semantic meaning over a larger context, allowing the notion of a differential diagnosis and multiple component symptoms to synergistically, rather than independently, influence the output of the model.

**Figure 3:**
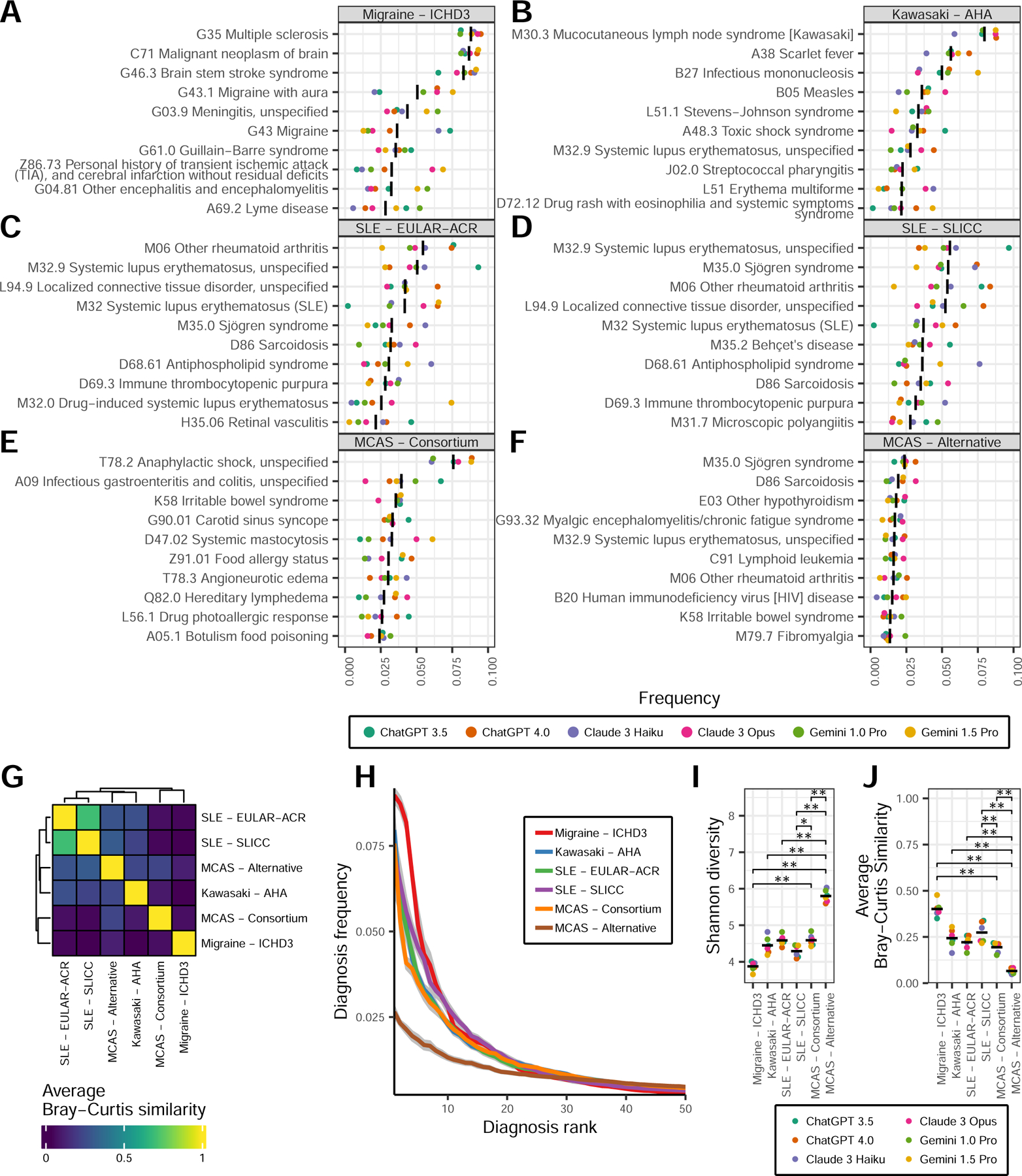
Diversity and precision of diagnoses associated with diagnostic criteria. Diagnosis distributions generated by repeated iterations of LLM queries using symptoms from each set of criteria. **A-F)** Frequencies of the top 10 diagnoses associated with each indicated diagnostic criteria. **G)** Bray-Curtis similarity between all diagnosis frequencies from each criteria, averaged across all models. **H)** Average diagnosis frequency in order of rank for the top 50 diagnoses from each set of criteria. **I)** Shannon diversity for the distribution of all diagnoses from each criteria. **J)** Precision as represented by the mean Bray-Curtis similarity between all 10,000 differential diagnosis iterations from each criteria and model. For **A-F, I-J**, colors represent indicated LLM, black bars represent the mean value across all models. For **H**, grey ribbons represent ± 1 standard error. For **I-J**, Wilcox rank sum p-values (*) < 0.05, (**) < 0.01, adjusted for multiple comparisons. Only comparisons involving MCAS criteria are annotated.

Validating this approach, for each set of control criteria, the expected diagnosis was found within the top 10 observed diagnoses and most of the top 10 conditions represented the same class of disorder as the expected diagnosis (e.g., rheumatologic for SLE criteria) (Figure 3A-D). The top diagnosis associated with consortium MCAS criteria represents the most severe acute manifestation of mast cell activation, anaphylaxis (Figure 3E). The top 10 diagnoses also included systemic mastocytosis and IgE mediated food allergy. In contrast, the top 10 diagnoses associated with alternative MCAS criteria did not include any allergic or primarily histamine-mediated conditions, instead spanning several distinct disease categories including rheumatologic, endocrine, hematologic, and infectious (Figure 3F). The similarity of all diagnoses generated by the two MCAS criteria was low, particularly compared to the similarity between SLE criteria (Figure 3G). Mast cell related conditions were ranked notably lower in the alternative MCAS criteria compared to the consortium MCAS criteria (Table 1). Together, this indicates significant dissimilarity in the types of conditions recognized by the two MCAS criteria, with alternative MCAS criteria more likely to overlap with conditions that are not primarily mast-cell mediated.

The top 50 diagnoses of the control conditions and consortium MCAS criteria accounted for the majority of each criteria’s total diagnoses (range 75.6 – 89.4%; Figure 3H). By comparison, only 48.4% of all alternative MCAS-associated diagnoses were found in the top 50. Correspondingly, the Shannon diversity of alternative MCAS criteria diagnoses was significantly higher than consortium MCAS criteria diagnoses (5.8 vs. 4.6, respectively; p-value=0.004), as well as all control criteria (p-value < 0.05 for all; Figure 3I). The consistency of each of the 10,000 differential diagnosis iterations within each set of criteria was significantly lower for alternative MCAS criteria compared to consortium MCAS criteria (Bray-Curtis similarity 0.07 vs 0.19, respectively; p-value=0.004) and control criteria for other diseases (all p-values < 0.01; Figure 3J). Collectively, these results show that alternative MCAS criteria overlap with a highly variable range of possible diagnoses and, compared to control and consortium MCAS criteria, are less able to reduce the range of diagnostic possibilities down to a narrow and consistent set of likely conditions.

**Figure 4:**
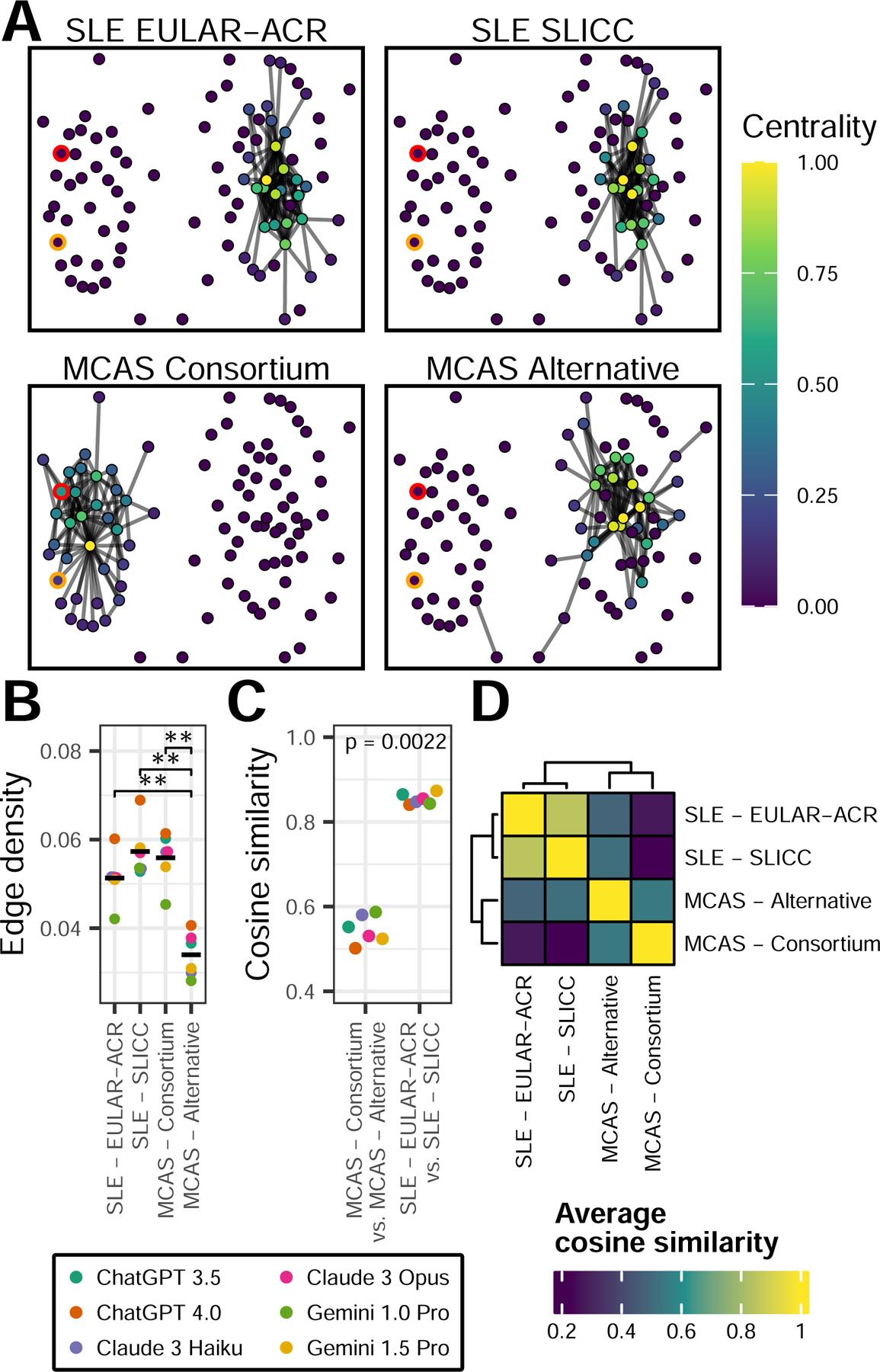
Network of co-occuring diagnoses associated with diagnostic criteria. **A)** Partial co-diagnosis graph. Nodes represent diagnoses. Edges are drawn between nodes if a pair is within the top 100 co-occuring diagnoses for the indicated criteria. Only diagnoses found within the top 100 co-occuring diagnoses of at least one set of criteria are drawn as nodes. Nodes colored by standardized eigenvalue centrality among all diagnoses for a given criteria. Red node represents mastocytosis, orange node represents mast cell activation syndrome. **B)** Mean edge density of complete co-diagnosis graph for each criteria. **C)** Cosine similarity for the centrality values of all nodes between the complete graphs of the indicated criteria. P-value by Wilcox rank sum test. **D)** As in **C** but averaged across all models to show similarity between all criteria networks. For **B-C**, colors represent indicated LLM, p-values by Wilcox rank sum test, (**) p-value < 0.01, adjusted for multiple comparisons.

Next, we hypothesized that, when modeled as a network, diagnostic criteria with a concise and reproducible differential diagnosis would generate a dense graph of co-occurring diagnoses. Indeed, the two sets of SLE criteria resulted in co-occurrence networks that shared a similar, dense structure (Figure 4A-B). By comparison, while the consortium MCAS criteria resulted in a similarly dense network, the topology of the alternative MCAS diagnosis network was more diffuse (edge density 0.06 vs 0.03, respectively; p-value=0.04). Based on the centrality of each diagnosis in the associated networks, the cosine similarity of the two MCAS criteria networks was significantly lower than the similarity between the two SLE criteria networks (0.55 vs. 0.86, respectively; p-value=0.0022) (Figure 4C-D). These results demonstrate that, compared to the observed similarity between two distinct sets of SLE criteria, consortium and alternative MCAS criteria diverge substantially in the range and types of conditions they can be associated with.

It is important to consider that a focus on pathophysiology-centered disease classification can contribute to stigmatization of patients presenting with non-specific symptoms lacking mechanistic explanation^19^. Patients with MCAS already report high rates of perceived stigmatization^20^ and the mechanism implied by the disease name likely provides validation to those affected by the chronic, non-specific symptoms included in the alternative MCAS criteria. As such, delabeling individuals overdiagnosed by alternative MCAS criteria risks worsening this sense of stigmatization.

However, inaccurate application of diagnostic labels that directly imply a specific pathophysiology can obscure appropriate management. For instance, the proposed mechanism for systemic sclerosis initially focused on skin exposure due to its original name, scleroderma^21^. Despite reports connecting scleroderma to silica as early as 1914, it wasn’t until the description of Erasmus syndrome in 1957, linking scleroderma and pulmonary disease in miners, that the condition was reframed as a systemic disease. Thus, bias imparted by the name scleroderma may have delayed identification of the causative link between inhaled silica and systemic sclerosis by 40 years. Similarly, overdiagnosis of MCAS by alternative criteria may delay identification of an appropriate mechanism and therapy for patients that do not meet consortium criteria. The likelihood that the alternative MCAS criteria unifies conditions with unrelated pathophysiologies may also explain the use of non-mast cell-directed therapies by its authors for patients that do not respond to typical therapy.

An additional ECNM-AIM designation, “MCAD unspecified,” represents a distinct clinical entity, marked by less severe presentations and less certainty about the relationship between symptoms and MCA^7^. Importantly, the ECNM-AIM recommendations emphasize that “unspecified MCAD” should not be considered a final diagnosis and should motivate further investigation for an underlying etiology. The alternative MCAS definition resembles the ECNM-AIM designation of “MCAD unspecified,” but utilizes the label of “MCAS” as a definitive diagnosis. Our analysis supports the ECNM-AIM recommendations that patients whose presentation matches “MCAD unspecified” or the alternative MCAS definition should be evaluated further to determine a more appropriate definitive diagnosis.

Arguably, a limitation of LLMs is that their training data represents a wide range of public text sources and is not restricted to medical literature. However, studies have confirmed that LLMs are capable of approximating high-level, open-ended, clinical reasoning^22^. Moreover, as most MCAS referrals are based on patient self-evaluation and internet research^12^, the broad training data underlying LLMs likely captures the symptom-diagnosis associations that lead patients to suspect MCAS. Additionally, the identification of expected diagnoses from the control criteria in our study supports the validity of using LLMs to simulate symptom-diagnosis associations.

In summary, we utilized multiple LLMs to bootstrap probability distributions for possible diagnoses resulting from different sets of MCAS criteria. We demonstrated that the broad range of symptoms included in the alternative MCAS criteria overlap with significantly more variable and less consistent diagnoses compared to consortium MCAS and control criteria. These results suggest that the proposed alternative MCAS criteria are an outlier in their relative lack of diagnostic specificity and their increased use risks overdiagnosis of MCAS to the exclusion of more appropriate diagnoses.

## Supplementary Material

1

## Figures and Tables

**Table 1:** Select diagnosis ranks. Rank of indicated diagnoses based on mean frequency within the consortium MCAS and alternative MCAS criteria-associated diagnoses distributions. Values in brackets represent individual ranks from the diagnosis distributions of ChatGPT 3.5, ChatGPT 4.0, Claude 3 Haiku, Claude 3 Opus, Gemini 1.0 Pro, and Gemini 1.5 Pro, respectively. NA reflects diagnoses not generated by the indicated model.

Diagnosis	MCAS - Consortium	MCAS - Alternative
T78.2 Anaphylactic shock, unspecified	1 [1, 1, 1, 1, 1, 1]	115 [215, 87, 99, 173, 139, 77]
D47.02 Systemic mastocytosis	5 [22, 8, 7, 2, 14, 2]	41 [92, 78, 25, 41, 46, 19]
D89.41 Monoclonal mast cell activation syndrome	29 [128, 22, 28, 11, 179, 51]	42 [141, 64, 22, 37, 168, 12]
D89.49 Other mast cell activation disorder	142 [307, 62, 101, 139, 475, 315]	383 [1139, 563, 178, 465, 1092, 275]
D89.4 Mast cell activation syndrome and related disorders	443 [690, 174, NA, NA, 588, 469]	1572 [NA, 812, 1720, 1458, 1726, 914]

## KEY MESSAGES

- Compared to consensus criteria, proposed alternative definitions of mast cell activation syndrome (MCAS) incorporate a significantly wider range of signs of symptoms, creating the potential for overdiagnosis.
- We utilize the intrinsic representations of clinical language within large language models (LLMs) in a novel approach to model the specificity of signs and symptoms included in diagnostic criteria.
- Probability distributions derived from LLMs demonstrate that diagnoses associated with alternative MCAS criteria are less specific to mast cell activation and less internally consistent compared to diagnoses associated with consortium MCAS criteria.

## ABBREVIATIONS

LLM: Large language model
MCA: Mast cell activation
MCAD: Mast cell activation disorders
MCAS: Mast cell activation syndrome
SLE: Systemic lupus erythematosus
