# Supplementary material for: Clustering of clinical symptoms using large language models reveals low diagnostic specificity of proposed alternatives to consensus mast cell activation syndrome criteria
Source: J Allergy Clin Immunol. Author manuscript; Available in PMC 2025 Jan 6. (PMC11700772; doi:10.1016/j.jaci.2024.09.006)
Supplement: 1 [file NIHMS2030915-supplement-1.pdf]

SUPPLEMENTAL METHODS

California inpatient ICD data

Inpatient diagnosis code data from 2005 to 2022 was obtained from https://data.chhs.ca.gov/dataset/hospital-inpatient-diagnosis-procedure-and-external-cause-codes. ICD code descriptions were standardized across years, while maintaining the distinction between pre- and post- ICD-10 code descriptions. Designations for mast cell activation-related conditions are only available starting in 2016 since no specific ICD codes existed for these conditions prior to ICD-10.

LLM embeddings

For LLMs to process language, words must first be converted into numerical representations. Word embedding models serve to convert words into dense numerical vectors where the distance between these vectors reflect semantic similarity between words. We utilized 4 advanced embedding models including OpenAI's text-embedding-3-small, Voyage AI's voyage-2-large, Google's text-embedding-004, and Mistral's mistral-embed. Using each model's provided API, we extracted embeddings for each of the symptoms listed in each set of diagnostic criteria (**Supplemental table 1**). Since each model generates embedding vectors of different lengths and clinical symptoms represent only a small range of the total word-embedding space of each model, we reduced the dimensionality and maximized the variability of the resulting embeddings using principal component analysis (PCA). Centroids for each set of diagnostic criteria were calculated by averaging the principal component embeddings of all symptoms in that set of criteria. Cosine similarity was used to represent the similarity between centroid embedding vectors, where 1=identical, 0=unrelated, and -1=opposite.

LLM differential diagnosis simulation

We utilized the following LLMs to generate diagnosis probability distributions: OpenAI's gpt-3.5-turbo-1106 and gpt-4-turbo-preview, Anthropic's claude-3-haiku-20240307 and claude-3-opus-20240229, and Google's gemini-1.0-pro-002 and gemini-1.5-pro-001. These models were chosen due to the availability of web interfaces that allow our results to be reproduced non-programmatically. Default parameters, including temperature, were used for all models. The following prompt was submitted to each LLM to generate a list of simulated differential diagnoses: "*For educational purposes, return a json list of format {{'diagnoses':[diagnosis list]}} with the top 10 diagnoses for the following combination of symptoms: {symptom_list}"}.*" Here, {symptom_list} was generated by randomly sampling 5 symptoms associated with a given set of diagnostic criteria. To ensure that the randomized symptoms were balanced across clinical systems (e.g. cardiovascular, neurologic, dermatologic, etc.), 5 systems were randomly selected with replacement first, followed by selection of a single random symptom from each of these systems to arrive at the final list of 5. For each set of criteria, **Supplemental table 1** lists the possible systems that are sampled first and then the list of possible symptoms that are sampled from each system. The hierarchy of systems and symptoms is taken directly from the cited sources for each set of criteria. This process of randomization and query assembly was repeated 10,000 times and the collection of all 10,000 randomizations can be found in the data/criteria_query_iterations.csv file in the associated GitHub repository (see below). Each query iteration was submitted through each model's API and the resulting diagnoses were parsed and tallied to generate a differential diagnosis distribution for each set of clinical criteria. Because 10 diagnoses were requested for each iteration, the maximum possible frequency of any diagnosis in the dataset was 10%.

Standardization of LLM output diagnoses to ICD codes

LLMs do not produce standardized diagnosis names, so variations for the same disorder are present in the summarized data both within and between models (e.g. SLE vs. systemic lupus erythematosus vs. lupus). To allow direct comparison between models, we standardized LLM output diagnoses to ICD codes. We downloaded the April 2024 version of ICD-10-CM from https://www.cdc.gov/nchs/icd/Comprehensive-Listing-of-ICD-10-CM-Files.htm, obtained ChatGPT `text-embedding-3-small` embeddings for all ICD code descriptions, and then reduced the dimensionality of these embeddings by PCA as described previously. ChatGPT embeddings in the same ICD code PCA space were similarly obtained for the output diagnoses of all LLMs. Each of these diagnoses was then matched to the closest ICD code based on its k-nearest neighbor (kNN) in the ICD code PCA embedding space (**Supplemental table 2)**.

Statistical analysis

All analyses were conducted in R/4.0.4. The Vegan/2.5 package was used to obtain Shannon diversity from a given differential diagnosis distribution and the Bray-Curtis similarity between iterations of 10-item differential diagnoses. All P-values were obtained using the Wilcox rank sum test with the Benjamini-Hochberg correction for multiple comparisons.

Network analysis

Network analysis and visualization was conducted using igraph/1.3.2, tidygraph/1.2.0, and ggraph/2.1.0. Nodes represent diagnoses and edges connect co-occurring diagnoses. Two diagnoses co-occur when they both appear in the output of a single iteration of a given LLM query (i.e., a single top 10 diagnosis list). For visualization, edges were filtered based on the frequency of diagnosis co-occurrence, as indicated. With the exception of diagnoses that only occurred a single time across all 10,000 iterations, the complete unfiltered networks were used for all calculations of network metrics. Node centrality was determined using eigenvalue centrality and the similarity between diagnostic criteria was calculated as the cosine similarity of all node centrality values for each diagnosis across the two associated networks. Edge density represents the ratio of observed edges to the total number of possible edges in the network.

Data and code availability

All LLM output data and analytic code necessary to reproduce these results is available at https://github.com/BenSolomon/mcas_criteria.

criteria	system	symptom
aha_kawasaki	constitutional	fever
	oral changes	lip erythema, lip cracking, tongue erythema, oropharyngeal erythema
	conjunctivitis	conjunctivitis
	rash	maculopapular rash, diffuse erythematous rash, erythema multiforme
	acral	hand edema, hand erythema, foot edema, foot erythema, periungual desquamation
	lymphadenopathy	cervical lymphadenopathy
	hematologic	anemia, thrombocytosis, leukocytosis
	renal	pyuria
eular_acr_sle	constitutional	fever
	hematologic	leukopenia, thrombocytopenia, autoimmune hemolysis
	neuropsychiatric	delirium, psychosis, seizure
	mucocutaneous	alopecia, oral ulcers, annular rash, psoriasiform rash, discoid rash, erythematous-violaceous, atrophic scarring, dyspigmentation, follicular hyperkeratosis, malar rash, maculopapular rash
	serosa	pleural effusion, pericardial effusion, acute pericarditis
	musculoskeletal	synovitis, arthropathy
	renal	proteinuria, nephritis
mcas_alternative	Constitutional	Fatigue, hyperthermia, hypothermia, sweats, flushing, plethora, pallor, increased appetite, decreased appetite, weight gain, weight loss, migratory pruritus, chemical sensitivities, physical sensitivities, poor healing
	Dermatologic/integument	Dermatographism, rashes, migratory patchy macular erythema, telangiectasias, angiomata, xerosis, striae, warts, tags, folliculitis, ulcers, dyshidrotic eczema, angioedema, alopecia, onychodystrophy
	Ophthalmologic	Irritated eyes, dry eyes, difficulty focusing vision, blepharospasm
	Otologic/osmic	Otitis externa, otitis media, hearing loss, tinnitus, dysosmia, coryza, post-nasal drip, congestion, epistaxis
	Oral/oropharyngeal	Oral pain, leukoplakia, ulcers, angioedema, dysgeusia, periodontal disease
	Lymphatic	Lymphadenopathy, lymphadenitis
	Pulmonary	Sinusitis, laryngitis, bronchitis, pneumonia, cough, dyspnea with normal pulmonary function tests, wheezing, obstructive sleep apnea
	Cardiovascular	Presyncope, postural orthostatic tachycardia syndrome, hypertension, blood pressure lability, palpitations, migratory edema, chest pain, atherosclerosis, takotsubo cardiomyopathy, Kounis syndrome, vascular anomalies
	Gastrointestinal	Dyspepsia, gastroesophageal reflux, nausea, vomiting, diarrhea, constipation, gastroparesis, angioedema, dysphagia, bloating, migratory abdominal pain, malabsorption
	Genitourinary	urinary tract infection, interstitial cystitis, chronic kidney disease, endometriosis, chronic back pain, infertility, decreased libido, vulvodynia, vaginitis, dysmenorrhea, menorrhagia, miscarriage

criteria	system	symptom
	Musculoskeletal/connective tissue	Bone pain, joint pain, muscle pain, hypermobility, osteopenia, osteoporosis, cysts, fibrosis, hemorrhoids, aneurysms, arteriovenous malformations
	Neurologic	Headache, sensory neuropathies, episodic weakness, dysautonomias, seizure disorders, pseudoseizures, cognitive dysfunction, memory problems, difficulty concentrating, word-finding difficulty, insomnia, frequent waking, hypersomnolence, non-restorative sleep, restless legs
	Psychiatric	Depression, irritability, mood lability, anxiety, obsessive-compulsive behavior, attention deficit, hyperactivity
	Endocrinologic/metabolic	Abnormal electrolytes, abnormal liver enzymes, hypothyroidism, hyperthyroidism, dyslipidemia, hyperglycemia, hypoglycemia, glycemic lability, hypoferritinemia, hyperferritinemia, delayed puberty
	Hematologic/coagulopathic	Polycythemia, anemia, leukocytosis, leukopenia, monocytosis, eosinophilia, basophilia, thrombocytosis, thrombocytopenia, thromboembolic disease, easy bruising, easy bleeding,
	Immunologic	Hypersensitivity reactions, increased susceptibility to infection, increased immunoglobulins, decreased immunoglobulins
mcas_consortium	cutaneous	flushing, pruritus, hives, angioedema
	gastrointestinal	vomiting, abdominal cramps, diarrhea
	respiratory	dyspnea, laryngeal edema, wheezing, hypoxia, nasal congestion, sneezing, rhinorrhea, conjunctival injections
	cardiovascular	hypotension, syncope, incontinence
migraine	headache	headache
	headache associated	nausea, vomiting, photophobia, phonophobia
	visual	scintillation, scotoma
	sensory	paresthesia, numbness
	speech	aphasia, dysphagia
	motor	weakness
	brainstem	vertigo, dysarthria, tinnitus, ataxia, diplopia, hearing loss
slicc_sle	acute cutaneous	malar rash, bullous rash, toxic epidermal necrolysis, maculopapular rash, photosensitive rash, psoriasiform rash, annular polycyclic rash, postinflammatory dyspigmentation, telangiectasias
	chronic cutaneous	discoid rash, verrucous rash, panniculitis, chilblain, lichen planus
	oral ulcers	oral ulcers, nasal ulcers
	nonscarring alopecia	nonscarring alopecia
	synovitis	synovitis, arthropathy
	serositis	pleural effusion, pericardial effusion, pericarditis
	renal	proteinuria
	neurologic	seizures, psychosis, mononeuritis multiplex, myelitis, peripheral neuropathy, cranial neuropathy, delirium
	hemolytic anemia	hemolytic anemia
	leukopenia	leukopenia, lymphopenia
	thrombocytopenia	thrombocytopenia

Supplemental table 1: List of all published diagnostic criteria utilized in this study.

ICD diagnosis	Original LLM outputs
D47.02 Systemic mastocytosis	angioedema associated with systemic mastocytosis • gastrointestinal mastocytosis • lupus mastocytosis • mastocytosis • mastocytosis (cutaneous and systemic) • systemic basophilic granulocytosis • systemic mastocytosis • elderly onset systemic mastocytosis • mast cell disorders (such as systemic mastocytosis) • mastocytosis (including cutaneous and systemic types) • mastocytosis (including cutaneous and systemic) • mastocytosis (including systemic mastocytosis and cutaneous mastocytosis) • mastocytosis (including systemic mastocytosis) • mastocytosis (systemic mastocytosis) • mastocytosis (systemic or cutaneous) • mastocytosis (systemic) • systemic mast cell disease • bullous mastocytosis • indolent systemic mastocytosis • fungal pneumonia with systemic mastocytosis • mast cell disorders (e.g., systemic mastocytosis) • mastocytosis with eosinophilia and otitis • mastocytosis with systemic involvement • mastocytosis with systemic symptoms • acute mastocytosis • generalized mastocytosis • genetic syndromes that involve mastocytosis, such as gorham-stouts disease or mccune-albright syndrome • hereditary mastocytosis • hypothetical, systemic mastocytosis (icd code d820). see 42820 • masocytosis • masto mastocytosis • mastocytosis (mc) • mastocytosis and systemic anaphylaxis, idiopathic • mastocytosis with an associated hematological neoplasm • mastocytosis with an associated solid tumor • mastocytosis with angioedema and syncope • mastocytosis with angioedema, syncope, rhinorrhea, and incontinence • mastocytosis with angioedema, vomiting, sneezing, and rhinorrhea • mastocytosis with associated flushing, vomiting, and laryngeal edema • mastocytosis with dysautonomia, hypersomnia and angioedema • mastocytosis with flushing • mastocytosis with flushing, hypotension, and conjunctival injection • mastocytosis with gastrointestinal involvement • mastocytosis with hypotension and angioedema • mastocytosis with syncope • mastocytosis with syncope and flushing • mastocytosis with urticaria, angioedema, and hypotension • mastocytosis, • mastocytosis, indolent systemic • mastocytosis, systemic • mastocytosis, systemic, with pruritis, vomiting, and diarrhea • mastocytosis: systemic (urticaria pigmentosa and extracutaneous mastocytosis, including mast cell leukemia) • systemic mastocytosis (sm) • systemic mastocytosis with leukocytosis and angiomata • justification: can cause similar gastrointestinal symptoms, but nasal congestion would be unusual., systemic mastocytosis • justification: systemic mastocytosis is a rare disorder characterized by the accumulation of mast cells in various tissues, leading to a variety of symptoms, including abdominal cramps, diarrhea, hives, angioedema, and in some cases, incontinence., anaphylaxis • mastocytosis (a rare disorder) • mastocytosis (consider if recurrent) • mastocytosis (e.g., systemic mastocytosis) • mastocytosis (e.g., systemic mastocytosis, cutaneous mastocytosis) • mastocytosis (rare) • mastocytosis (rare, but can cause all these symptoms) • mastocytosis (systemic mast cell activation disorder) • mastocytosis (systemic mastocytosis, cutaneous mastocytosis)

Supplemental table 2: Representative mapping of original LLM output diagnoses to a standardized ICD code based on kNN matching of PCA-reduced embedding vectors.
